# Supplementary material for: Highly energetic phenomena in water electrolysis
Source: Sci Rep. 2016 Dec 16;6:39381. doi: 10.1038/srep39381 (PMC5159792; doi:10.1038/srep39381)
Supplement: Supplementary Information [file srep39381-s4.pdf]

**SUPPLEMENTARY INFORMATION**

# Highly energetic phenomena in water electrolysis

A. V. Postnikov,[1] I. V. Uvarov,[1] M. V. Lokhanin,[2] and V. B. Svetovoy [1, 3, *]

[1]*Yaroslavl Branch of the Institute of Physics and Technology,*
*Russian Academy of Sciencies, 150007 Yaroslavl, Russia*
[2]*P. G. Demidov Yaroslavl State University, Sovetskaya 14, 150000 Yaroslavl, Russia*
[3]*MESA+ Institute for Nanotechnology, University of Twente, PO 217, 7500 AE Enschede, The Netherlands*

## Supplementary files

1. Sound file Sound.wav corresponds to the driving pulses $U = 6.75\ V$ and $f = 150\ kHz$. The PCB-device was fixed at the bottom of a plastic Petri dish of 10 cm in diameter.
2. Video file V1.avi was taken at 100000 fps with the driving pulses $U = 6.75\ V$ and $f = 150\ kHz$.
3. Video file V2.avi was taken at 54000 fps with the driving pulses $U = 6.75\ V$ and $f = 150\ kHz$. Appearance and termination of a bubble smaller than in V1.avi can be seen.

**Supplementary figures**

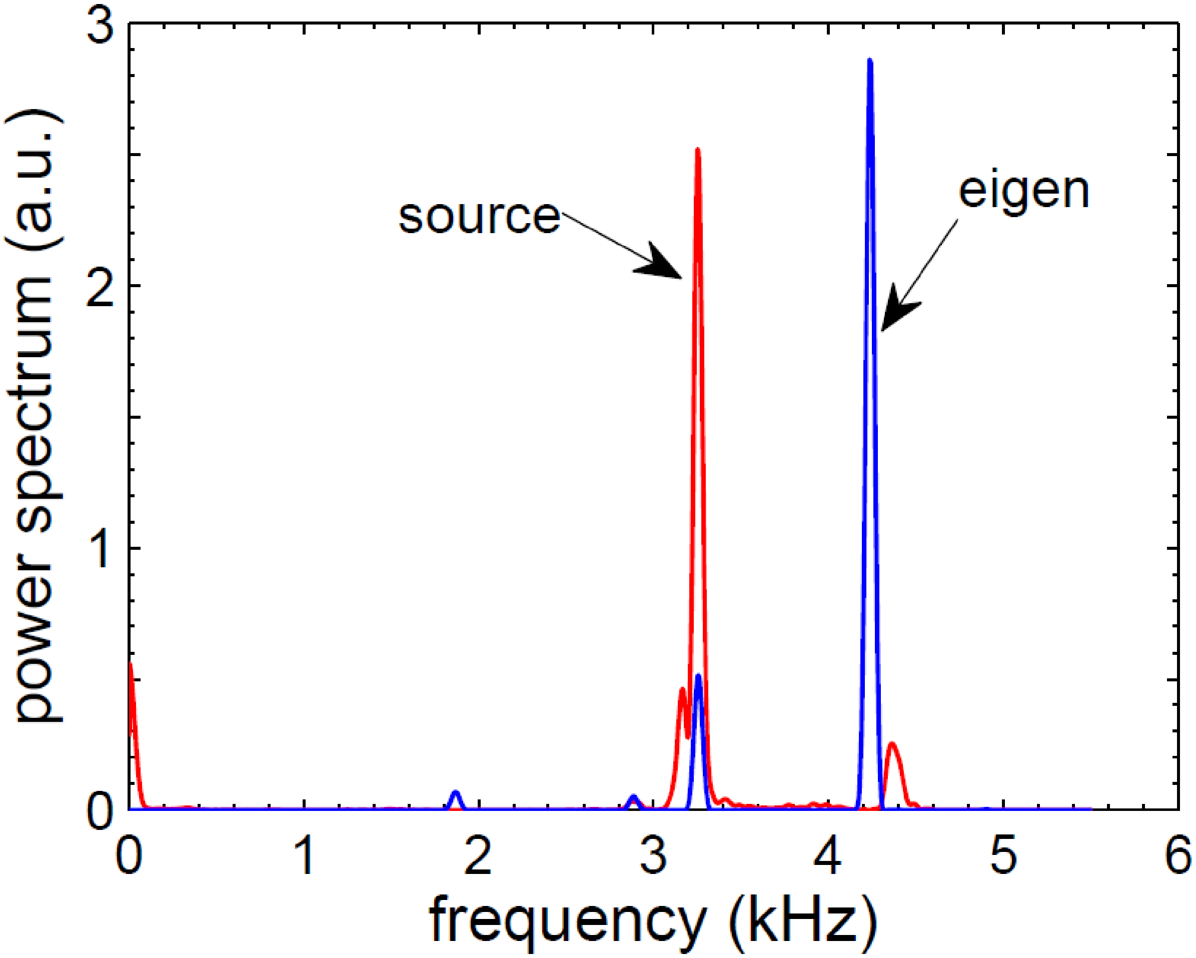


**Figure 1S**. Frequency composition of the sound for the experiment performed in a glass dish. The red curve corresponds to the spectrum of the sound produced by the PCB-device (source). The blue curve presents the spectral composition of the sound produced by a gentle hit on the dish edge (eigen).

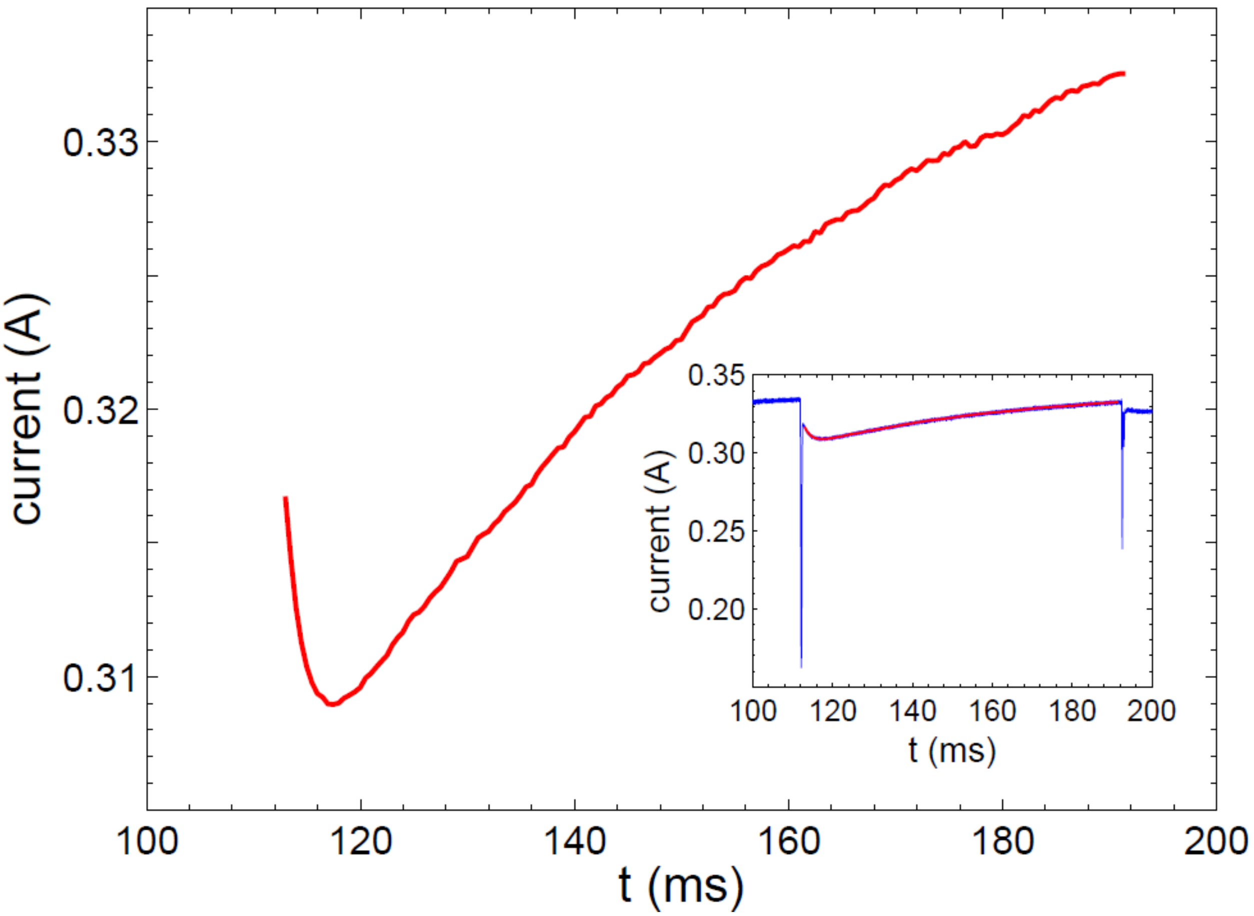


**Figure 2S.** Long-time dynamics of the current. The inset shows the upper part of the enveloping line for the current between two clicks. The clicks correspond to the one shown by the arrow and the next one in Fig. 2 in the text. The red curve in the inset and in the main graph is the current (enveloping line) between the two clicks averaged over 5 $\mu$s. First, the current is reduced since colder liquid comes to the electrodes after the bubble termination. Due to Joule heating and due to the effect of the reaction this liquid is heated up and the current increases with time. The current is related to the temperature change as $I = I_0(1 + \alpha\Delta T)$, where $I_0$ is the current at $\Delta T = 0$ and $\alpha \approx 0.024\ K^{-1}$. Using the graph one can estimate the temperature change as $\Delta T \approx 3^\circ\ K$. The time scale for the heating is defined by $\tau \sim a^2/\kappa = 70\ ms$, where $a = 100\ \mu m$ is the radius of the central electrode, where the current density is the highest, and $\kappa = 1.4 \times 10^{-7}\ m^2/s$ is the heat diffusion coefficient in water.
